# Acute Ventricular Wall Thickening: Sepsis, Thrombotic Microangiopathy, or Myocarditis?

**DOI:** 10.1155/2015/275825

**Published:** 2015-03-15

**Authors:** Nicolas De Schryver, Delphine Hoton, Diego Castanares-Zapatero, Philippe Hantson

**Affiliations:** ^1^Department of Intensive Care, Cliniques Universitaires Saint-Luc, Université Catholique de Louvain, 1200 Brussels, Belgium; ^2^Department of Pathology, Cliniques Universitaires Saint-Luc, Université Catholique de Louvain, 1200 Brussels, Belgium

## Abstract

*Background*. Acute myocardial oedema has been documented in experimental models of ischemia-reperfusion injury or sepsis and is usually investigated by magnetic resonance imaging. *Purpose*. We describe a case of acute ventricular wall thickening documented by echocardiography in a patient developing sepsis and thrombotic microangiopathy. *Case Description*. A 40-year-old woman, with a history of mixed connective tissue disease, was admitted with laryngeal oedema and fever. She developed *Streptococcus pneumoniae* septicaemia and subsequent laboratory abnormalities were consistent with a thrombotic microangiopathy. Echocardiography revealed an impressive diffuse thickening of the whole myocardium (interventricular septum 18 mm; posterior wall 16 mm) with diffuse hypokinesia and markedly reduced left ventricular ejection fraction (31%). There was also a moderate pericardial effusion. Echocardiography was normal two months before. The patient died from acute heart failure. Macroscopic and microscopic examination of the heart suggested that the ventricular wall thickening was induced by oedematous changes, together with an excess of inflammatory cells. *Conclusion*. Acute ventricular wall thickening that corresponded to myocardial oedema as a first hypothesis was observed at echocardiography during the course of septicaemia complicated by thrombotic microangiopathy.

## 1. Introduction

The rapid development of ventricular thickening at echocardiography is suggestive of interstitial oedema rather than muscular hypertrophy. Common causes of interstitial oedema are myocarditis and ischemia-reperfusion phenomena. While experimental evidence suggests that septic cardiomyopathy may also be associated with interstitial oedematous changes, few data appeared in human cases in the acute phase of severe sepsis. We discuss the possible mechanisms of acute ventricular thickening in a complex case of combined septicaemia, thrombotic microangiopathy (TMA), and mixed connective tissue disease (MCTD).

## 2. Case Description

A 40-year-old Black African woman presented to the hospital for rapid onset of facial swelling and dysphagia. Her medical history included a mild form of MCTD diagnosed in 2006 (Sharp's syndrome with anti-DNA and anti-RNP antibodies), three previous episodes of angioneurotic oedema, two recent episodes of septicaemia (*Streptococcus agalactiae *and* pneumoniae*), and a tuboovarian abscess treated by laparoscopic drainage 2 months before.

Her daily treatment included hydroxychloroquine 400 mg/d, nebivolol 5 mg/d, calcium carbonate 1.25 g/d, and esomeprazole 20 mg/d. On admission, she complained of facial swelling with laryngeal stridor and fever measured at 39.8°C. Clinical examination revealed heart rate 119 bpm, blood pressure 190/90 mmHg, respiratory rate 16/min, and O_2_ saturation (pulse oximetry) 94%. Cardiopulmonary auscultation was unremarkable as was abdomen examination. Laboratory investigations showed C-reactive protein 0.7 mg/dL (N < 1.0 mg/dL), serum creatinine 0.56 mg/dL, sodium 138 mmol/L, potassium 3.05 mmol/L, lactate dehydrogenase (LDH) 296 IU/L (N < 248 IU/L), hemoglobin 10.5 g/dL, white blood cell count 6740/*μ*L, and platelet count 158 000/*μ*L. Urinalysis failed to reveal leukocyturia or bacteriuria, and chest X-ray examination was normal. She was immediately administered 1 g methylprednisolone intravenously (i.v.) and 1 g tranexamic acid i.v., together with aerosol therapy, but her clinical condition rapidly deteriorated with a worsening of laryngeal stridor. She became hypoxemic and finally required orotracheal intubation and mechanical ventilation. Antimicrobial therapy with ceftazidime was initiated after taking blood cultures. Endoscopic laryngoscopy ruled out epiglottitis. At contrast-enhanced abdominal computed tomography (CT), there was no recurrence of tuboovarian abscess or other obvious sources of infection. Corticosteroids were continued at the daily dose of 1 g methylprednisolone.

**Figure 1:**
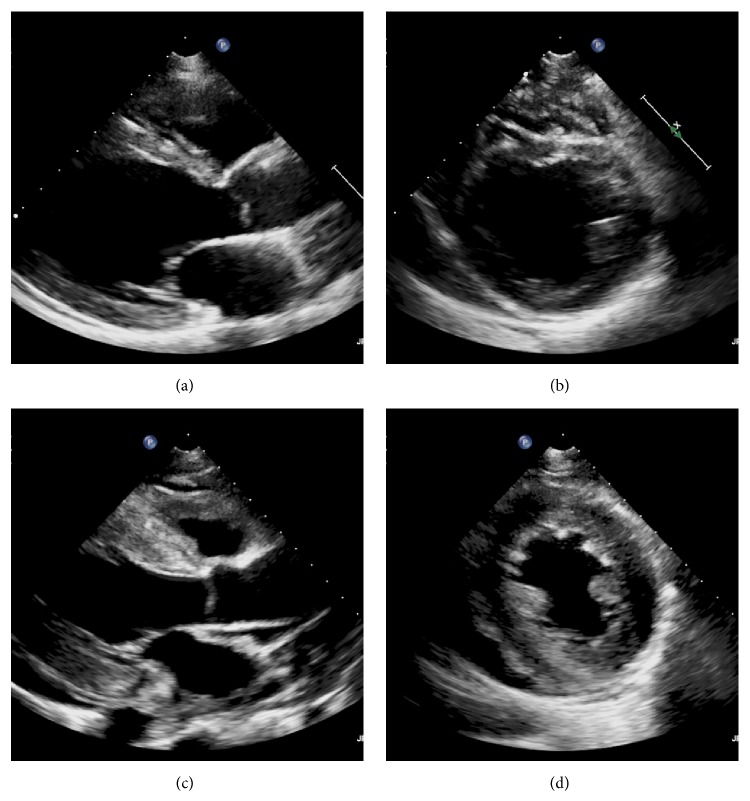
Telediastolic parasternal long (panel (a)) and short (panel (b)) axis view showing normal thickness of the myocardium 2 months before admission. Panels (c) and (d) show telediastolic parasternal long and short axis view, respectively, of the same patient, 6 days after admission. Impressive diffuse myocardium thickening is observed.

**Figure 2:**
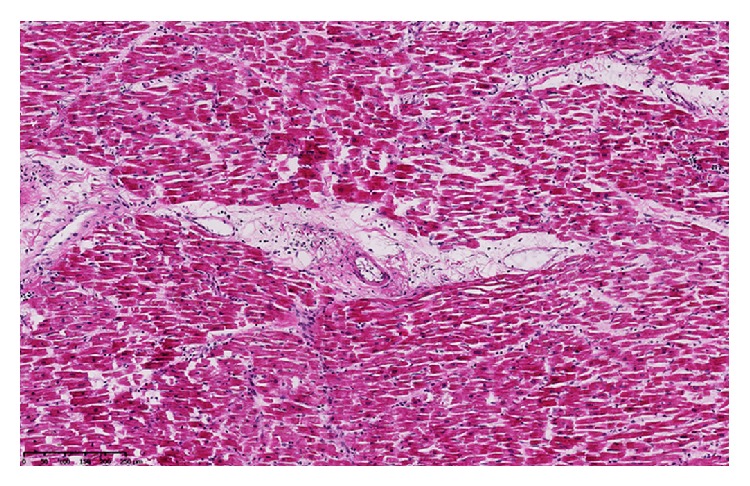
Ultrastructural examination of the myocardium showing diffuse interstitial oedema with mononuclear cells inflammatory infiltration.

**Figure 3:**
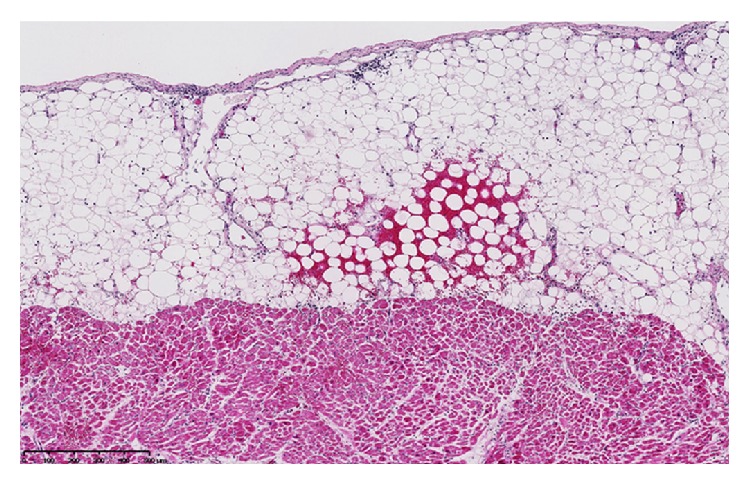
Ultrastructural examination of the pericardium showing rare hemorrhagic spots with a slight infiltration of inflammatory cells.

The next days, a marked thrombocytopenia appeared (platelets 18,000/*μ*L on day 4 and 9,000 on day 5) with signs of hemolysis (LDH 570 IU/L on day 4, schistocytes 4% on day 5 and 7% on day 6). Haptoglobin was <10 mg/dL (N 30–200 mg/dL) on day 5. A thrombotic microangiopathy (TMA) was suspected. Anti-heparin/platelet factor 4 antibodies were undetectable. Admission blood cultures grew for* Streptococcus pneumoniae* and ceftazidime was replaced by ceftriaxone. Plasma exchange was considered. To evaluate filling pressures, a transthoracic echocardiography was performed on day 6. Surprisingly, it revealed an impressive diffuse thickening of the whole myocardium (interventricular septum 18 mm; posterior wall 16 mm) with diffuse hypokinesia and markedly reduced left ventricular ejection fraction (31%) (Figure 1). Left ventricle end-diastolic and end-systolic diameters were 41 mm and 36 mm, respectively. A moderate pericardial effusion (9 mm) was also observed without hemodynamic changes. There was no increase in cardiac biomarkers. Importantly, an echocardiography had been performed 2 months before and was unremarkable. At this time, cardiac output still seemed satisfactory with normal diuresis, normal lactate level, and no need for inotropic or vasopressor support. Several hours later, she suddenly presented electromechanical dissociation with cardiac arrest before initiation of plasma exchange. Cardiopulmonary resuscitation was immediately initiated and pericardial puncture was performed in the same time but removed less than 10 mL serohemorrhagic fluid. Spontaneous circulation could not be restored and the patient was declared dead. An autopsy was performed. No source of infection was found. A small pericardial effusion was confirmed (210 mL of serohemorrhagic fluid). Macroscopic examination of the heart revealed diffuse thickening of the myocardium. Ultrastructural examination confirmed oedematous thickening of the myocardium with an excess of inflammatory cells (mononuclear and polymorphonuclear cells) and small hemorrhagic spots (Figure 2). No vascular thrombus or myocardial infarction was observed. No amyloid substance deposit was shown with red Congo staining. Examination of the pericardium revealed slight infiltration by lymphoid cells (Figure 3). Protease ADAMTS13 activity was determined postmortem and was undetectable in the blood sample drawn before cardiac arrest (N > 40). No inhibitor of the protease ADAMTS13 was detected suggesting that the deficit was hereditary. Common viral causes of myocarditis were excluded by extensive laboratory investigations.

## 3. Discussion

We report a fatal case of ventricular wall thickening caused by myocardial oedema in the course of an episode of TMA possibly induced by a* Streptococcus pneumoniae* septicaemia in a patient with a MCTD.

Myocardial oedema (MO) has been widely observed by magnetic resonance imaging (MRI) in various cardiac conditions involving ischemia-reperfusion phenomena like myocardial infarction, Takotsubo syndrome, cardiopulmonary bypass, heart transplantation, or resuscitation [1]. It correlates on pathological examination with cardiomyocytes swelling and interstitial oedema. Mechanisms involved are increased vascular permeability induced by inflammatory mediators (VGEF, MMP, and thrombin) causing endothelial contraction, osmotic modification in the ischemic region, and Na^+^ overload of the myocytes and platelet activation. MO has also been described in several conditions other than ischemia-reperfusion. These include increased afterload and inflammation associated with sepsis, viral myocarditis, or autoimmune diseases. Pathological heart examination of patients dying from septic shock frequently may reveal mononuclear infiltrates and interstitial oedema but the specificity of these observations is probably very low [2]. In the early phase of sepsis, MO has been poorly described and only in animal models where it has been shown to precede cardiac dysfunction [3, 4]. Moreover, in this setting, involved mechanisms seem to be different as it incriminates interendothelial cell junction damages [5].

MO is best detected by MRI, but this examination cannot be safely performed in critically ill patients and is usually replaced by bedside echocardiography. The most common echocardiographic features are however not specific for MO. Experience is mostly available from acute myocarditis, where an acute change in ventricular wall thickness is reflecting oedema formation and cell infiltration [6]. Some authors have indeed reported that echocardiographic tissue characterization of the myocardium using ultrasonic backscatter could be helpful to investigate changes in myocardial density and elasticity that could occur in myocarditis [7, 8]. These data are unfortunately not available in our patient. However, we believe that the acute onset of left ventricular hypertrophy at echocardiography was a valuable sign suggestive of MO. This finding can be compared to acute left ventricular hypertrophy due to massive MO that has been described in animal models within the first minutes of reperfusion after 90 min occlusion of a major epicardial vessel [9]. A similar echocardiographic feature due to acute myocardial oedema formation has also been reported following the autonomic storm of brain death [10].

In our patient, MO may have been induced by several factors including connective tissue disease and sepsis. Whether TMA played a role in the observed cardiac involvement remains questionable. Both* Streptococcus pneumoniae* septicaemia and MCTD have been associated with TMA [11, 12]. Several cardiac events associated with thrombotic microangiopathy have already been observed in rare case reports or cohort studies [13]. These include myocardial infarction, congestive heart failure, and arrhythmias. Reduced left ventricular function, wall motion abnormalities, pericardial effusion, and tamponade are echocardiographic findings occasionally described. Autopsies with ultrastructural examination of the heart reported presence of thrombi in arterioles and capillaries, myocardial hemorrhage, myocardial infarction, or endocarditis [13]. Surprisingly, we observed in our patient an impressive myocardial thickening with decreased left ventricular ejection fraction that correlated with ultrastructural examination showing an oedematous infiltration of the myocardium. No histologic sign was found that suggested a cardiac involvement caused by TMA. The infiltration of inflammatory cells with presence of pericardial effusion could also correspond to a certain degree of myocarditis that may have progressed despite prolonged administration of corticosteroids. The true prevalence of cardiac involvement in MCTD is not precisely known, as most patients remain poorly symptomatic [14]. Pericardial effusion is the most frequent echocardiographic sign, but myocardial thickening is not mentioned in the patients who developed biopsy-proven acute fulminant myocarditis [14, 15].

## 4. Limitations

Several other points remain unclear. First, while this episode of TMA seemed to be related to infection and/or to MCTD, the origin of* Streptococcus pneumoniae* septicaemia was not found, even at autopsy. Second, the sudden onset of electromechanical dissociation without any previous sign of low cardiac output was surprising, even if nonischemic acute heart failure has been exceptionally reported in TMA patients [16]. This seemed to be induced by the rapid worsening of cardiac oedema, rather than by the pericardial effusion that remained limited. Third, as plasma exchange could not be initiated before the patient's deterioration, it is not known whether it could have improved the outcome.

## 5. Conclusions

We report the case of a young woman who developed fatal acute cardiac oedema in the course of an episode of TMA after a* Streptococcus pneumoniae* septicaemia. Whether this cardiac involvement was directly associated with sepsis, TMA, or a certain degree of myocarditis is unclear. Clinicians should recognize this pattern mimicking hypertrophic cardiomyopathy as a probable marker of poor prognosis in case of sepsis or TMA.
